# Near-field focus steering along arbitrary trajectory via multi-lined distributed nanoslits

**DOI:** 10.1038/srep33317

**Published:** 2016-09-13

**Authors:** Gun-Yeal Lee, Seung-Yeol Lee, Hansik Yun, Hyeonsoo Park, Joonsoo Kim, Kyookeun Lee, Byoungho Lee

**Affiliations:** 1 National Creative Research Center for Active Plasmonics Application Systems, Inter-University Semiconductor Research Center and School of Electrical and Computer Engineering, Seoul National University, Gwanak-Gu Gwanakro 1, Seoul 08826, Korea

## Abstract

The modulation of near-field signals has recently attracted considerable interest because of demands for the development of nano-scale optical devices that are capable of overcoming the diffraction limit of light. In this paper, we propose a new type of tuneable plasmonic lens that permits the foci of surface plasmon polariton (SPP) signals to be continuously steered by adjusting the input polarization state. The proposed structure consists of multi-lined nanoslit arrays, in which each array is tilted at a different angle to provide polarization sensitivity and the nanoslit size is adjusted to balance the relative amplitudes of the excited SPPs from each line. The nanoslits of each line are designed to focus SPPs at different positions; hence, the SPP focal length can be tuned by modifying the incident polarization state. Unlike in previously reported studies, our method enables plasmonic foci to be continuously varied with a smooth change in the incident linear polarization state. The proposed structures provide a novel degree of freedom in the multiplexing of near fields. Such characteristics are expected to enable the realization of active SPP modulation that can be applied in near-field imaging, optical tweezing systems, and integrated nano-devices.

Surface plasmon polaritons (SPPs) are surface electromagnetic waves propagating along a metal-dielectric interface that are coupled with the oscillation of electrons^1^,^2^,^3^. In the past two decades, SPPs have attracted extensive interest because they offer the possibility of manipulating light on a subwavelength scale. Various applications have been proposed, including chemical and biological sensors^4^,^5^, near-field holographic imaging systems^6^,^7^,^8^, nano-scale particle tweezing^9^,^10^ and integrated photonic devices^11^,^12^,^13^.

In particular, the synthesis and control of SPPs using metal films and nano-structures have been of interest to numerous researchers for the use of SPPs in practical applications. Hence, plasmonic devices that generate optical beaming, near-field focusing, and vortex fields have been successfully demonstrated^14^,^15^,^16^,^17^,^18^,^19^,^20^. Most of these advances have made use of SPPs that are excited from nanoslits on a metal film. It is known that the SPPs generated from nanoslits can be modelled as electric or magnetic dipole sources that are overlaid on a metal surface^21^, which makes them quite amenable to SPP field manipulation because of their highly predictable excitation phase.

Recently, considerable efforts have been made to dynamically tune plasmonic foci by modifying various external conditions, such as the incidence angle of light^22^,^23^, geometric changes in a fluidic lens^24^, the angular momentum numbers of an incident Laguerre–Bessel beam^25^,^26^,^27^,^28^,^29^, and external signals with phase change materials^30^,^31^. However, tuning methods based on the incidence angle of light suffer from a need for highly accurate optical path alignment and are difficult to implement because of the need to rotate the overall sample. Structures based on geometric changes in a fluidic lens have low reproducibility and thus are not suitable for practical applications. Plasmonic tuneable structures based on the angular momentum number are applicable only for specific types of incident beam, such as Bessel beams. Moreover, such structures require additional optical devices such as spatial light modulators to generate Bessel beams with arbitrary angular momentum numbers, which leads to a bulky optical system. Phase change materials are specific materials whose optical properties depend on external signals such as an electric current or thermal condition. Using phase change materials is also a useful method of realizing tuneable devices. However, their performance is subject to certain limitations with regard to the realization of continuously tuneable devices; only a partial range of phases can be modulated, or it is difficult to continuously vary the external signal.

One of the possible methods of overcoming the above issues and limitations is to use the optical polarization of the incident light for SPP tuning. Circularly or linearly polarized light has been used to control plasmonic foci. For example, various investigators have used circular polarization states to switch the direction of SPP propagation^32^,^33^,^34^,^35^ or to produce a spatial phase distribution of SPPs^36^. In previous research, we reported that SPP wavefronts can be freely designed for incident light with right- or left-circular polarization (RCP or LCP) by using line-envelope nanoslit arrays^36^. However, in the previously reported work, only two states were used for wavefront expression. Two distinct focal points were pre-determined for RCP and LCP incidence, and it was not possible to continuously steer these focal points. For continuous steering of an SPP focal point, we applied a strategy based on linear polarization instead of circular polarization. Using a continuous set of angles of linear polarization states is suitable for the complex tuning of SPPs in nanoslit-based structures because a linear polarization has a clear directionality for each polarization state. More recently, efforts have been made to use linear polarization for tuneable plasmonic lenses. Lerman and Levy switched a near-field focus by illuminating a pin-cushion-like slit with linearly polarized light^37^. Wintz *et al*. reported on 4-port multiplexing achieved by combining two polarization angles and two wavelengths^38^. To the best of our knowledge, however, no research has been reported in which the continuous tuning of the foci of plasmonic lenses has been achieved by using omnidirectional linear polarization angles.

In this paper, we propose tuneable plasmonic lenses that continuously control the foci of SPPs by changing the incident linear polarization state. The basic principle of the proposed structure relies on the polarization-sensitive characteristics of nanoslits. The nature of SPPs excited from nanoslits is related to the orientations and distribution of the nanoslits. Hence, we use multi-lined nanoslit arrays to continuously steer the plasmonic focus. Each line consists of a nanoslit array with a fixed tilt angle, which is selected to excite the maximal SPP in a certain polarization state. Lateral shifts of the nanoslits generate the SPP focus and determine the focal length for a specific incident polarization state. By means of these accurate designs, the SPP focus can be continuously steered along an arbitrary trajectory with the continuous rotation of the linearly polarized incident light, as the line that dominantly excites the SPPs changes. To confirm the performance of the proposed structure, numerical calculations based on the dipole modelling method, the finite element method (FEM), and finite-difference time-domain (FDTD) simulations were performed. We also fabricated the proposed structure on a silver film and measured the near-field profile using near-field scanning optical microscopy (NSOM).

## Results

**Figure 1:**
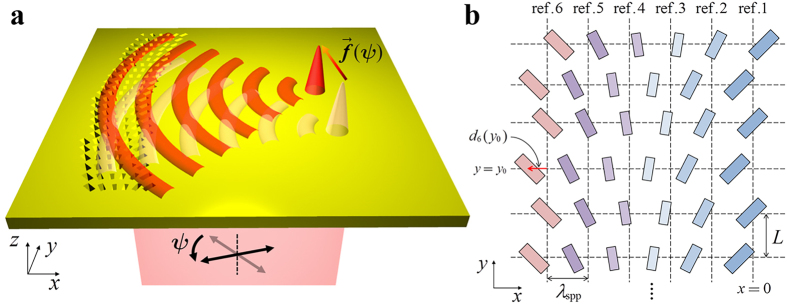
(**a**) Schematic diagram of the proposed tuneable plasmonic lens. When linearly polarized light with polarization angle 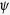 is normally incident on the nanoslits, SPPs are excited and propagate towards the focal point, the position of which is determined by the pattern of the nanoslit arrays. The focal point moves along the trajectory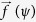 as the polarization angle 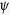 varies, and the magnitude and the trajectory of the focal spot are determined by the size, tilt angle, and position of the nanoslits. (**b**) Detailed top-view schematic of the nanoslit array. Along the *y* axis, the nanoslits are separated by a distance *L*. There are 6 equidistant reference lines (ref. 1~^6^), which are parallel to the *y* axis, and the interval between these reference lines is equal to the effective SPP wavelength (*λ*_spp_). The position *x* = 0 is defined as the *x* position of the rightmost reference line. Each nanoslit has a different size and tilt angle and is shifted by *d*_*i*_(*y*) with respect to the reference lines.

A schematic diagram of the proposed plasmonic lens is shown in Fig. 1a. The structure consists of several lines of nanoslit arrays on a silver film. When a light beam is normally incident on the back side of the structure, the nanoslit arrays generate SPPs and the superposition of the SPPs generated from all nanoslits propagates along the metal surface. For the formation of a plasmonic focus, the nanoslits are laterally shifted to form a parabolic phase distribution along the *y* direction. As previously mentioned, our goal is to be able to continuously tune the plasmonic focus by changing the incident polarization state. To achieve this behaviour, it is necessary to be able to control the amplitude of the SPPs excited from each line by varying the incident polarization state.

### Principles and numerical calculations

In Fig. 1b, we show a detailed top view of the proposed structure. There are six reference lines along the *y* axis, and the nanoslits are distributed side by side around the reference lines. The nanoslits belonging to the *i*^th^ line have a tilt angle 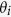 with respect to the *y* axis and a size 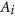. Here, 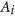 is defined as a size factor that represents the relative amplitude ratio of the excited SPPs due to the size of the nanoslit. A different size is needed to compensate for the amplitude of the SPPs excited by each line with a different tilt angle, and a detailed analysis of the slit size and excited amplitude is presented below. The interval between the reference lines is set to the effective wavelength of the SPPs 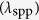, which is given by 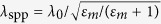, where 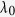 and 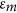 are the free-space wavelength of the incident light and the permittivity of the metal; these values are set to 980 nm and −43.9 + 2.77*i*, respectively, throughout this paper^37^. The parameter *L* is the distance along the *y* axis between nanoslits in the same line. Here, *L* is set to 400 nm, which is smaller than half of the effective SPP wavelength (*λ*_spp_ = 968 nm); thus, we can fully express the designed phase profile without in-plane diffraction^32^.

The nanoslits in each line are shifted by some distance along the *x* axis from the reference line. The distance of this shift is determined by the desired location of the plasmonic focus at the target incident polarization for that line. The target incident polarization for each line is determined by its tilt angle 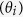; thus, the SPP propagation will be sensitive to the incident polarization state. As a proof of the proposed concept, we consider two examples throughout this paper, namely, the continuous tuning of the plasmonic focus along the *x* and *y* axes.

**Figure 2:**
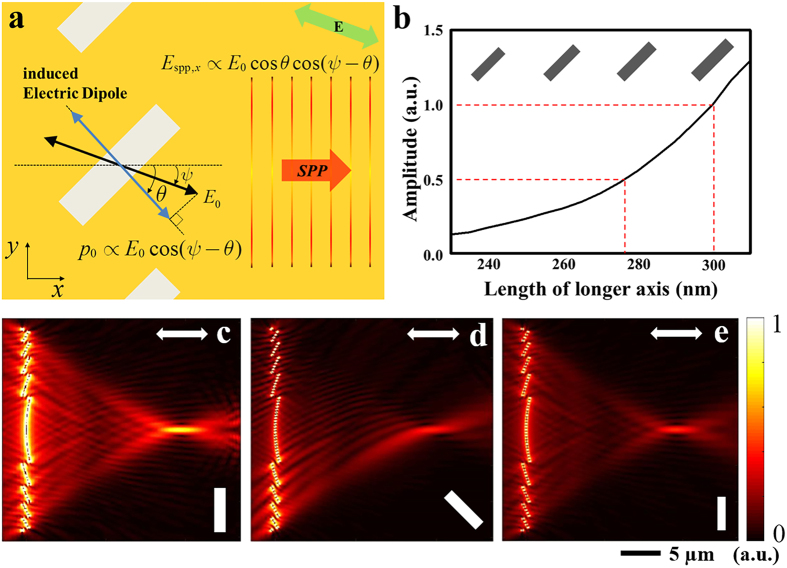
(**a**) Schematic diagram for explaining the dependence of the amplitude of the excited SPPs on the tilt angle of the nanoslit array. (**b**) Amplitude of the SPPs generated from a nanoslit as a function of the length of the longer axis. (**c**–**e**) Electric field amplitude 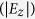 distributions in the *xy* plane for a single-line plasmonic lens when the longer length and tilt angle of the nanoslits of which it consists are respectively (**c**) 300 nm and 0°, (**d**) 300 nm and 45°, and (**e**) 277 nm and 0°. All of the designed plasmonic lenses focus the SPPs on the point (20 μm, 0 μm). The electric field amplitudes at the foci are (**c**) 0.0396 V/m, (**d**) 0.0202 V/m and (**e**) 0.0203 V/m. The electric field amplitude of the incident light is 1 V/m.

Here, we briefly explain the mechanism of the excitation of SPPs from a nanoslit array. As depicted in Fig. 2a, linearly polarized light, whose electric field orientation is rotated by 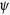 with respect to the *x* axis, illuminates the nanoslit array. When the tilt angle of a nanoslit with respect to the *y* axis is denoted by 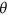, the amplitude of the SPPs excited from that nanoslit is proportional to 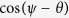
27. The reason for this is that only the component of the electric field that is perpendicular to the longer axis of the nanoslit can dominantly affect the generation of SPPs. The necessary condition is satisfied when the size of the nanoslit is at the subwavelength scale and the aspect ratio of the nanoslit is sufficiently high. In this study, the aspect ratio of the nanoslits is the same as that used in our previous work, namely, 4 by 1, which has previously been verified to satisfy the aforementioned condition^32^.

In addition to the 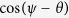 factor that originates from a single nanoslit, it is necessary to consider the interference of each SPP source on the metal surface. Because the nanoslits are arranged along the *y* direction, only the *x* components of the SPPs that are excited from each nanoslit interfere constructively. According to Huygens’ principle, SPPs propagate along the *x* direction with an additional amplitude factor of 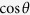. Therefore, the amplitude of the plane-wave SPPs generated from the array of tilted nanoslits is proportional to 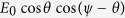, where *E*_0_ is the amplitude of the incident electric field.

This relation can also be directly derived from the previously reported work that reported complex amplitude relations for circularly polarized incident light^32^. According to the relation, we can calculate the complex amplitude for any linear polarization state as a linear combination of the RCP and LCP states (see Supplementary information, Part 1), as below:


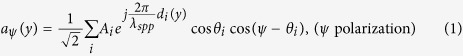


where *i* is the number of reference lines, 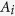 is the size factor, 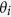 is the tilt angle, and 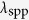 is the effective SPP wavelength. To create parabolic wavefronts of a plasmonic lens with focal position (*f*_*x*,*i*_, *f*_*y*,*i*_), the formula for the shift distance of the nanoslits becomes 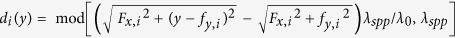, where 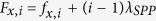 is used to compensate for the phase delay caused by the geometric separation of each line. Here, we confirm that the complex amplitude of the SPPs along the *x* axis is proportional to 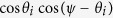. Of the two factors, the 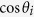 factor is more worthy of notice. To retain a wide range of possible foci, there must be a wide range of tilt angles. However, the 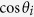 factor causes amplitude distortion; therefore, without compensation, the range of 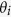 is restricted. As a compensation factor, it is necessary to adjust the size of the nanoslits in each line to produce SPPs of equal amplitude.

Figure 2b shows the amplitude of the SPPs excited from a nanoslit. The results were calculated using FEM-based software (COMSOL 5.0). The aspect ratio of the nanoslit was fixed at 4:1 to prevent variations in any SPP excitation characteristics other than the amplitude. The amplitude of the SPPs was observed at a fixed probe placed 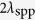 away from the centre of the nanoslit. In our demonstration, the range of the tilt angle was designed to run from *θ*_1_ = −π/4 to *θ*_6_ = π/4, which requires a compensation factor of two with respect to a zero-tilt nanoslit. It is possible to show that by changing the size of the nanoslit from 240 nm to 300 nm, the normalized amplitude of the excited SPPs can cover the range from 1/2 to 1, as is needed. The amplitudes of the excited SPPs shown in Fig. 2b are normalized with respect to that produced by a 300 nm × 75 nm nanoslit. We also confirm that the amplitude of the excited SPPs is proportional to the size of the nanoslit. Hence, the size factor can be easily designed within this range.

Figure 2c–e illustrate examples of plasmonic lenses with single-line nanoslit arrays before and after amplitude compensation. The field distributions were calculated by means of FDTD simulations. The white rectangular bars on the lower right side of each figure indicate the orientation and size of each nanoslit, and the white arrows on the upper right side represent the polarization state of the incident light. Figure 2c,d show the intensity distributions of SPPs generated from single-line plasmonic lenses composed of nanoslits of the same size (300 nm × 75 nm) but with different tilt angles of 0° and 45°, respectively. Because of the factor of cos^2^ 45° (corresponding to the 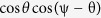 factor in equation (1)) in the light amplitude, the amplitude at the focus for the 45° case is theoretically half of that for the 0° case. When incident *x*-polarized light with an electric field amplitude of 1 V/m normally illuminates the back side of the structure, the calculated maximum amplitudes at the focus are 0.0396 V/m and 0.0202 V/m in Fig. 2c,d, respectively; these values are different by a factor of two, as predicted. For comparison, an example after amplitude compensation is shown in Fig. 2e. According to the relation illustrated in Fig. 2b, the amplitude of the SPPs excited from a 300 nm × 75 nm nanoslit is twice that of the SPPs excited from a 277 nm × 69.3 nm nanoslit. Therefore, the structure represented in Fig. 2e consists of a nanoslit with dimensions of 277 nm × 69.3 nm and a tilt angle of 0°. The maximum amplitudes at the focus in Fig. 2d,e are 0.0202 V/m and 0.0203 V/m, respectively. As expected, they are nearly identical, with an error of less than 1%. Therefore, it is possible to compensate for the tilt-angle effect such that the excitation amplitudes for nanoslit arrays with 0° and 45° tilt angles can be made equal, and an analogous compensation is applied to every line. With this compensation, equation (1) can be rewritten as follows:


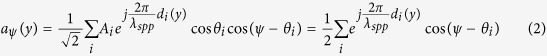


where 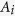 is set to satisfy 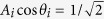. Using equation (2), the SPPs generated from each line can be equally combined.

### Application: a tuneable plasmonic lens

As proofs of concept for the proposed method, two types of plasmonic lenses were designed. Both plasmonic lenses generate SPP foci, but in one, the focus moves along the *x* axis when the incident polarization vector rotates, whereas in the other, it moves along the *y* axis. We named these structures *x*-control and *y*-control tuneable plasmonic lenses, respectively.

**Figure 3:**
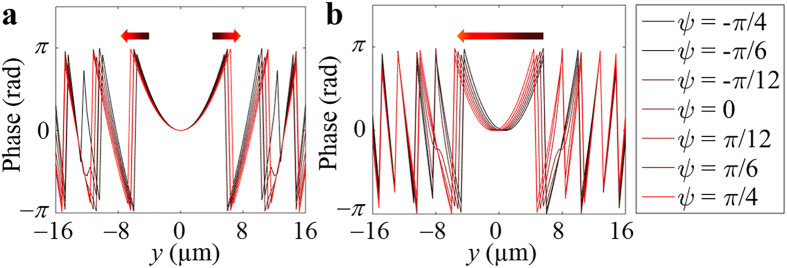
Spatial phase distributions of SPPs at *x* = 0 for (**a**) *x*-control and (**b**) *y*-control tuneable plasmonic lenses. The phase distribution of (**a**) becomes wider as the polarization angle *ψ* continuously changes from −*π*/4 to *π*/4, whereas the phase distribution of (**b**) shifts along the *y* axis under the same change in polarization angle.

Figure 3a,b show the phase distributions of the superposed SPPs for the proposed *x*-control and *y*-control tuneable plasmonic lenses, respectively, along the rightmost reference line. Each graph illustrates the tuneable characteristics of the plasmonic focus as the incident polarization angle 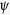 is changed from −*π*/4 to *π*/4, which are represented by graded-colour lines from black (*ψ* = −*π*/4) to red (*ψ* = *π*/4). In this study, the tuning range of the plasmonic focus was designed to be (*f*_*x*,1_, *f*_*y*,1_) = (29 μm, 0 μm) to (*f*_*x*,6_, *f*_*y*,6_) = (43 μm, 0 μm) for the *x*-control structure and (*f*_*x*,1_, *f*_*y*,1_) = (25 μm, 4 μm) to (*f*_*x*,6_, *f*_*y*,6_) = (25 μm, −4 μm) for the *y*-control structure. It can be shown in Fig. 3a that curvature of parabola becomes broader for the *x*-control, preserving the centre position. For *y*-control, in contrast, the curvature remains constant while the centre position shifts, as shown in Fig. 3b. There observations indicate that the focal position is shifted along the *x* and *y* direction, respectively. However, the far-centred phase distributions, which are related with the quality of foci, have some limitations to represent the clear change during polarization changes. This limitation is according to the number of reference lines of our structure.

The number of reference lines is an important variable in designing structures using the proposed method. In an ideal case, a larger number of reference lines are preferred to achieve a better quality of continuous modulation, but it results in a larger structure. Therefore, considering the physical constraints of device realization, additional compromise is unavoidable. To investigate the effect of the number of reference lines, *x*-control tuneable plasmonic lenses with different numbers of reference lines were analysed through numerical simulation (see Supplementary Information, Part 2). The simulation results show that a number of reference lines greater than 6 is sufficient for continuous variation of the complex field profile. Moreover, when the SPPs that are generated from one nanoslit array pass through the next nanoslit array, secondary scattering phenomena such as reflection and diffraction will occur, causing the amplitude of the SPPs excited from the back-side line to decrease. Increasing the number of reference lines results in more secondary scattering between the nanoslit arrays and increases the size of the overall structure. According to our FEM calculations, the amount of secondary scattering that occurs when plane SPP waves pass through a nanoslit array varies with the tilt angle of the array. By varying the tilt angle, the amount of secondary scattering that occurs upon passing through a nanoslit array was calculated to be approximately 8% on average (see Supplementary Information, Part 3). In other words, an excessive number of lines may degrade the tuning performance.

In this research, the optimal number of array lines was determined to be 6, in order to guarantee continuous change of the phase of the complex SPP fields without any significant reduction in power due to secondary scattering. The lowest SPP amplitude that is observed after the SPPs generated from the leftmost nanoslit array have passed through the other 5 nanoslit arrays is still approximately 71.3% of the original amplitude.

## Experimental results

**Figure 4:**
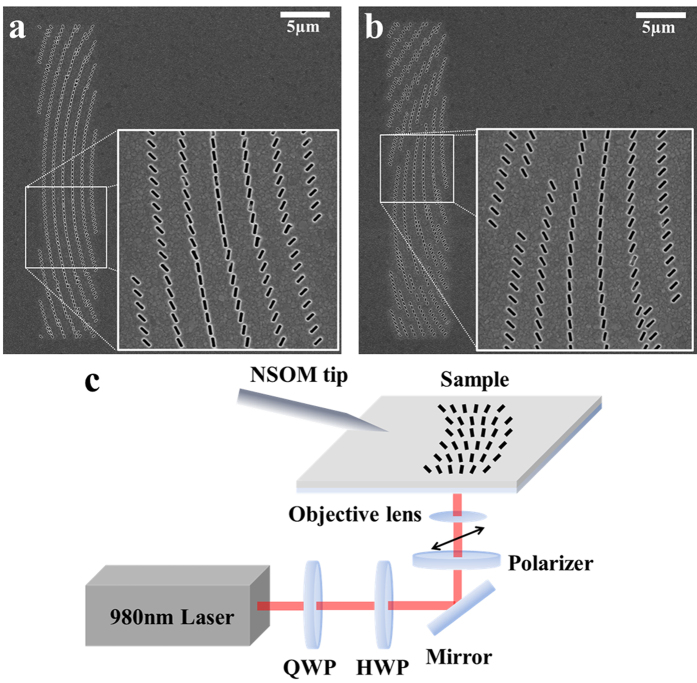
(**a**,**b**) FE-SEM images of fabricated (**a**) *x*-control and (**b**) *y*-control structures. The insets (white boxes) show detailed views of the slit patterns. (**c**) Schematic diagram of the NSOM measurement setup. The near-field distribution generated by the structure was collected through the aperture in the NSOM tip.

Figure 4a,b show field-emission scanning electron microscope (FE-SEM) images of fabricated samples that were designed as *x*-control and *y*-control tuneable plasmonic lenses, respectively. The insets present magnified views of the image regions within the white boxes.

The near-field profiles of the fabricated structures were then measured using NSOM (Nanonics, Multiview 4000), as shown in Fig. 4c. A near-infrared laser with a wavelength of 980 nm illuminated the back side of the silver film after passing through a quarter-wave plate (QWP), a half-wave plate (HWP), a mirror, and a linear polarizer. The QWP and HWP were used to set the laser beam into right-circular polarization, and the linear polarizer was then used to create a linear polarization state and rotate its angle. A polarimeter (Thorlabs, PAN5710IR) for an infrared source was used to confirm the polarization state.

**Figure 5:**
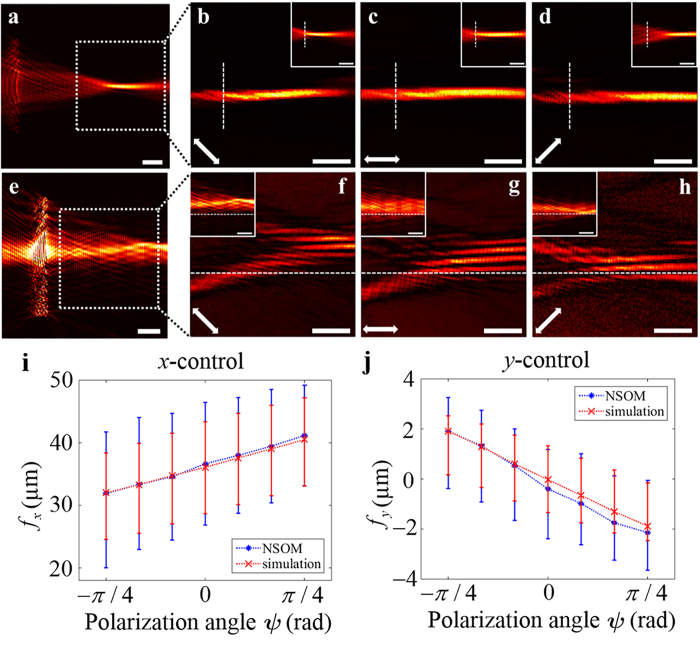
(**a**–**h**) Calculated and measured near-field distributions for (**a**–**d**) an *x*-control tuneable plasmonic lens and (**e**–**h**) a *y*-control lens. The length of the white bar in each figure is 7.5 μm. (**a**,**e**) Calculated near-field distribution for (**a**) *x*-control and (**e**) *y*-control structure. White boxes in the figures are sized as 33 μm by 33 μm. (**b**–**d**, **f**–**h**) The main figures represent the results measured using NSOM, and the results of numerical simulations based on dipole modelling are shown in the white boxes. The white arrows on the lower left side indicate the polarization of the incident light (−45°, 0°, or 45°). (**i**–**j**) The positions of the SPP foci versus the polarization angle 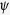 for the (**i**) *x*-control and (**j**) *y*-control structures. The red dotted lines with cross markers represent the results of the numerical simulations, and the blue dotted lines with circle markers correspond to the NSOM data. The lengths of the red and blue vertical bars represent full-width at half maximum (FWHM) of the simulated and experimental focus profiles for each case.

Figure 5 shows the simulation and experimental results for the near-field profiles for linear polarization states. Figure 5a,e show the simulation results for the near-field profiles of the *x*-control and the *y*-control tuneable plasmonic lenses including devices which are located on left side of the figures. Total areas of the figures are 61 μm by 61 μm for Fig. 5a and 55 μm by 55 μm for Fig. 5e. Due to limited scanning window of the NSOM system, 33 μm by 33 μm, only the field profiles near the foci are measured, instead of scanning the whole device. Figure 5b–d,f–h present the results for the *x*-control and the *y*-control tuneable plasmonic lenses, respectively, for three polarization states of −45°, 0° and 45°. The corresponding numerical simulation results are also presented for comparison in the white boxes in the upper right corners of each subfigure. The field profiles for other linear polarization states are shown in Movie S1, Movie S2, and Supplementary Information Part 4. The white scale bars in the figures represent a length of 7.5 μm, and the white arrows on the lower left side indicate the polarization state of the incident light. It can be clearly seen that the focal points move along either the *x* axis or *y* axis, as intended, in both the simulated and experimental results. For a more precise comparison, the positions of the foci at each polarization angle are plotted in Fig. 5i,j for the *x*-control and *y*-control structures, respectively. To determine the positions of the foci, we calculated the mean position value by performing a surface integral of the near-field distribution from either the simulated or experimental data. In the experimental process, we were able to find the absolute focal position values by using optical microscopy system with a charge-coupled device (CCD) camera and objective lens. We detected the spatial relationship between the sample and the NSOM tip, and we got the *xy* coordinate values of the sample and the NSOM tip from the NSOM software. By using these values, we recalculated the absolute focal position values *f*_*x*_ and *f*_*y*_ even if we only detected the partial area without the sample in the NSOM experiment. The red dotted lines with cross markers were obtained from the simulated data, whereas the blue lines with circle markers correspond to the NSOM data. Here, we also calculated full-widths at half maximum (FWHM) to show the focal sizes and accurately analyse movements of SPP focal area, and the results are also represented in Fig. 5i,j as vertical bars for each case. As intended, the focus positions exhibit a linear dependence on the angle of the incident polarization state. However, the sizes of the focal spots generated from the proposed structures are relatively large compared to single-lined plasmonic structures such as the structures illustrated in the Fig. 2c–e. This phenomenon is caused by the effect of multi-lined structures. The proposed structures consist of some single-lined plasmonic lenses of which focal positions are different from each other. According to the equation (2), for an arbitrary input polarization, not only the main reference line corresponding to the input polarization but also other reference lines near the main reference line generate weak SPP signals. Those SPP signals form the SPP foci whose positions are different to the position of the SPP focus generated from the main reference line, and it makes the focal spot broader. In other words, it means that the effective numerical aperture (NA) of the overall structure is determined by the NA values which are different from each other, and this phenomenon induces blurring of the focus.

Moreover, there are unexpected differences between the NSOM and simulated results near the π/4 polarization, as depicted on the right side of Fig. 5i. We conclude that the mismatch in these regions originates from secondary scattering from previous nanoslit arrays. As discussed above, the SPP field excited by incident light with a π/4 polarization is generated primarily from the π/4 reference line, which is the farthest from the focus. Therefore, the SPPs generated from this reference line are likely to be significantly influenced by secondary effects. Although we do not address this issue in this paper, we hope that the previously discussed compensation technique based on slit-size modification can also be applied to overcome this degradation due to secondary scattering by calculating the extent to which the amplitude decreases when the SPPs pass through each nanoslit array (see Supplementary Information, Part 2).

## Discussion

In summary, we theoretically discussed the phenomenon in which linearly polarized light illuminates a nanoslit with a given tilt angle and an arbitrary size, and we applied this understanding to modulate the complex amplitudes of SPPs generated from nanoslits. As a useful application of these properties, we designed, fabricated, and demonstrated tuneable plasmonic lenses whose foci continuously move on numerous micrometre-scale lines along the *x* axis or *y* axis as the incident linear polarization rotates through 90 degrees. Using simulations and NSOM experiments, we showed that the proposed structures generate SPPs from the illuminating light, focus them on an established focal area depending on the polarization state, and tune them within the micro-scale range.

This work can be easily extended to the continuous arbitrary tuning of a complex near field, controlled by the linear polarization state of the incident light. We conclude that the proposed scheme has the potential to be adapted for application in various fields of research, such as particle tweezing, the development of integrated nanodevices with plane waveguides, and near-field spatial light control.

## Methods

### Sample fabrication

For the fabrication of the proposed structures, a thin silver film with a thickness of 200 nm was deposited on a clean glass plate using an electron-beam evaporator (MUHAN, MHs-1800). Nanoslit arrays were then inscribed on the silver film using focused ion beam (FIB) equipment (FEI, Helios 650).

## Additional Information

**How to cite this article**: Lee, G.-Y. *et al*. Near-field focus steering along arbitrary trajectory via multi-lined distributed nanoslits. *Sci. Rep.* **6**, 33317; doi: 10.1038/srep33317 (2016).

## Supplementary Material

Supplementary Information

Supplementary Movie S1

Supplementary Movie S2
