# Supplementary material for: Near-field focus steering along arbitrary trajectory via multi-lined distributed nanoslits
Source: Sci Rep. 2016 Sep 13;6:33317. doi: 10.1038/srep33317 (PMC5020422; doi:10.1038/srep33317)
Supplement: Supplementary Information [file srep33317-s1.pdf]

Supplementary Information

Near-field focus steering along arbitrary trajectory via multi-lined distributed nanoslits

Gun–Yeal Lee, Seung–Yeol Lee, Hansik Yun, Hyeonsoo Park, Joonsoo Kim, Kyookeun Lee, and Byoungho Lee[*]

National Creative Research Center for Active Plasmonics Application Systems, Inter–University Semiconductor Research Center and School of Electrical Engineering, Seoul National University, Gwanak–Gu Gwanakro 1, Seoul 08826, Korea

*Corresponding Author's E–mail: Byuongho@snu.ac.kr

Part 1. Derivation of complex amplitude equation for linear polarization state

According to the theory of polarization, an arbitrary linear polarization state can be expressed as a combination of two orthogonal circular polarization states. Hence, a complex amplitude expression for linear polarization can be derived from the previously reported expression for circular polarization. Under the assumption of circularly polarized incident light, the relation is written as [1]

$$a_{R,L}(y) = \sum_i A_i \cos\theta_i e^{j\left(\frac{2\pi}{\lambda_{spp}} d_i(y) \pm \theta_i\right)} \quad \text{(RCP or LCP)} \tag{1}$$

where *i* is the number of reference lines, A_i is the size factor, θ_i is the tilt angle, d_i is the shift distance of the nanoslit, and λ_{spp} is the effective SPP wavelength. From eq 1, we can calculate the complex amplitude for any linear polarization state as a linear combination of the RCP and LCP cases, as below:

$$a_\psi(y) = \frac{1}{\sqrt{2}}\left(e^{j\psi} a_R(y) + e^{-j\psi} a_L(y)\right) = \frac{1}{\sqrt{2}} \sum_i A_i e^{j\frac{2\pi}{\lambda_{spp}} d_i(y)} \cos\theta_i \cos(\psi - \theta_i) \quad (\psi \text{ polarization}) \tag{2}$$

Part 2. Secondary scatterings of the nanoslit arrays

The diffraction and reflection of SPP due to scattering by neighboring nanoslits are discussed in this section. When surface plasmon polaritons (SPPs) propagating on the metal surface encounter obstructions such as nanoslits, diffraction and reflection will occur. Therefore, there must be diffractions and reflections at each line of the proposed multi-line metasurface structure. Here, the simplest example, diffraction and reflection characteristics of a plane SPP wave after passing through a nanoslit array is theoretically analyzed using the finite element method (FEM). Figure S1 shows the configuration of our full-field 3D simulation. In the simulation, SPPs

propagate on the silver surface along the *x* axis, and make contact with a tilted, 300 nm×75 nm sized nanoslit. The tilt angle is defined as the angle between the *y* axis and the longer axis of the nanoslit. Periodic boundary conditions are applied in the upper and lower boundaries, which implies that the simulated structure is the infinite length of the nanoslit array. We calculated the diffracted and reflected power for various values of tilt angle, since the scattering cross section of the tilted nanoslit with respect to the SPPs propagating in the *x*-direction depends on the tilt angle of the nanoslit. SPP propagation would be interrupted more with increasing scattering cross section; hence, reflection and diffraction are expected to have a maximum value when the tilt angle of the nanoslit is $0°$.

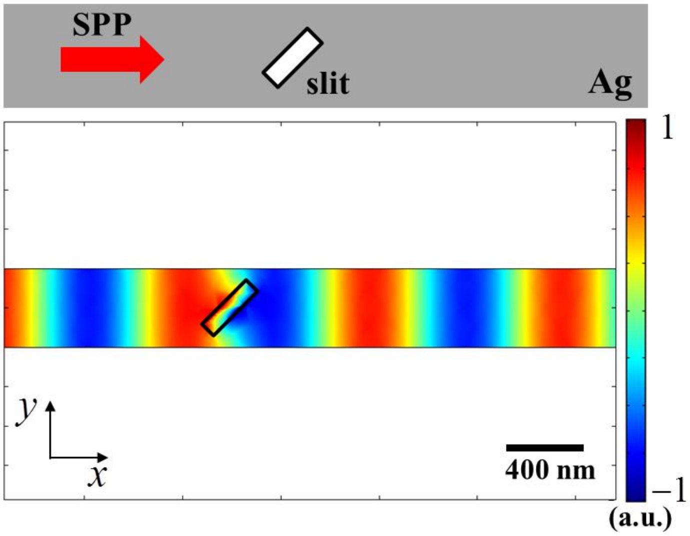

Figure S1. *z*-component of the electric field distribution when the SPP (λ_{spp} = 968 nm) is launched toward the nanoslit array from the left. The aspect ratio of the nanoslit is 300 nm×75 nm, and the tilt angle of the nanoslit is $45°$.

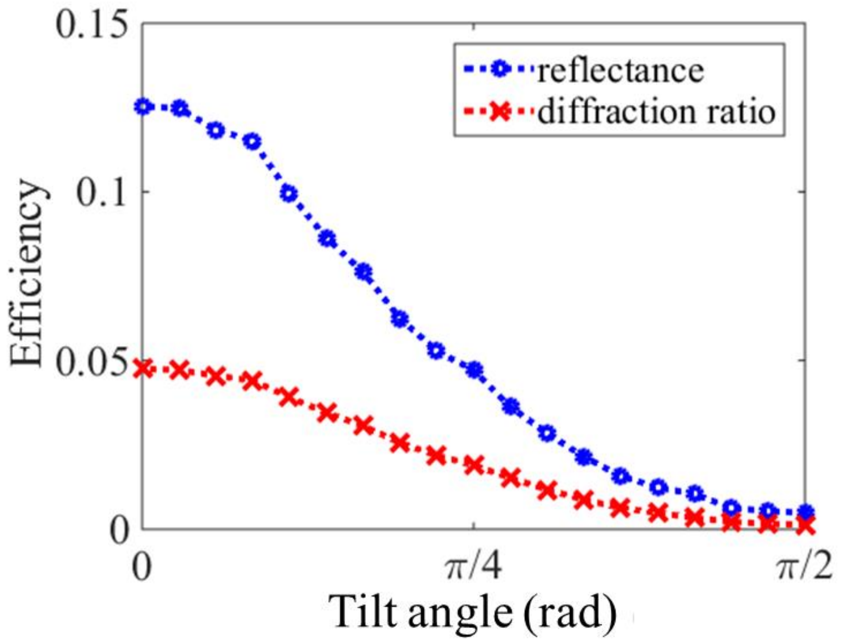

Figure S2. Normalized reflectance and diffraction ratio versus the tilt angle of nanoslit. The tilt angle is defined as the angle between the x axis and the longer axis of the nanoslit.

Figure S2 shows the reflectance and diffraction ratio as functions of the tilt angle of the nanoslits. Both the reflectance and the diffraction ratio decrease with increasing tilt angle of nanoslit from 0 to $\pi/2$, as we expected.

The mean values are roughly 0.055, and 0.022 for the reflectance and diffraction ratio, respectively. Therefore, about 8% of the input power would be lost due to secondary scatterings at the nanoslits. More specifically, the total decreased intensity ratios for specific tilt angles which were used to design the main structures are tilt angles of 16.6%, 11.5%, and 6.6% for $9°$, $27°$, and $45°$, respectively.

Part 3. Change in field distribution for some linear polarization angles versus the number of reference lines

From the eq 3, it can be expected that the dependence of the SPP profile on the polarization angle of the incident light would become smoother with increasing number of reference lines. Ignoring scattering and thermal losses, increasing the number of reference lines is desirable for controlling the complex amplitude profile via the polarization angle. However, employing more reference lines would lead to a more bulky structure and the scattering effects which are discussed in supplement Part 1 would become even more severe problems. Here, we analyzed the SPP profiles of the *x*-control plasmonic lens for different values of polarization angle and the number of reference lines (2-6 lines) as shown in Figure S3. In Figure S3, the rightmost reference line is at $x = -20$ μm. The compared structures are commonly designed to focus the SPPs into the ($f_{x,\mathrm{start}}$, $f_{y,\mathrm{start}}$) = (0 μm, 0 μm) at a $-\pi/4$ input polarization angle, and ($f_{x,\mathrm{end}}$, $f_{y,\mathrm{end}}$) = (10 μm, 0 μm) at a $\pi/4$ input polarization angle. The *x*-coordinate of the focal point changes continuously from $f_{x,\mathrm{start}}$ to $f_{x,\mathrm{end}}$ when the polarization angle changes from $-\pi/4$ to $\pi/4$. When there are only few reference lines, the focal point moves too abruptly or splits. In our case, we set the number of reference lines to 6 which allows the focal point to move smoothly.

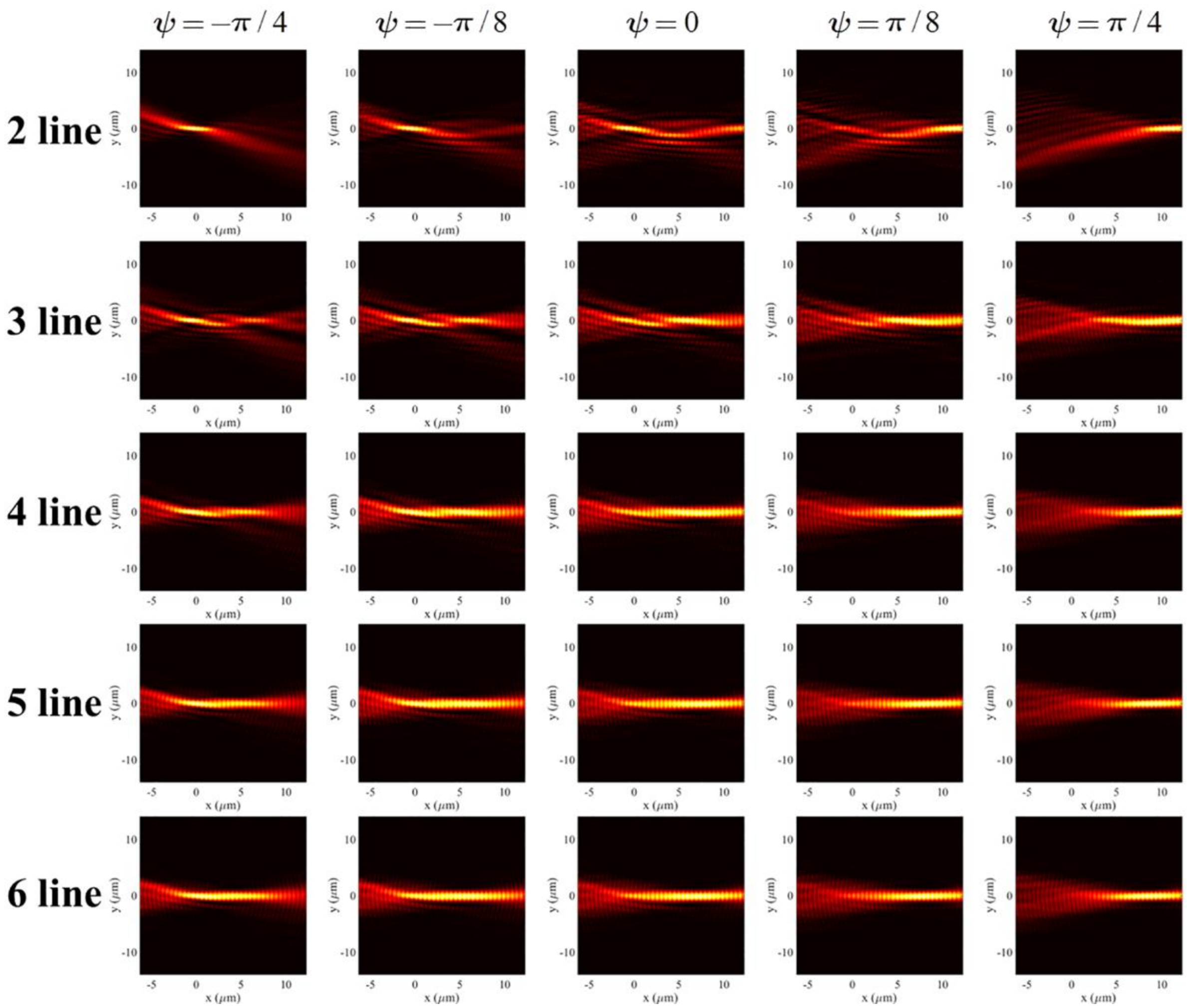

Figure S3. Calculated field distributions of structures with various number of reference lines at each polarization angle.

Part 4. Explanation on Movie S1 & S2

The *z*-component of the electric field distributions at each polarization is shown in Movie S1 and Movie S2. Movie S1 shows the field distributions of the *x*-control tunable plasmonic lens, and Movie S2 shows the field distributions for the *y*-control one. Each movie shows both results of numerical simulations and NSOM experimental data. As expected, the field distributions move continuously when the linear polarization vector of the illuminated beam is continuously rotated.

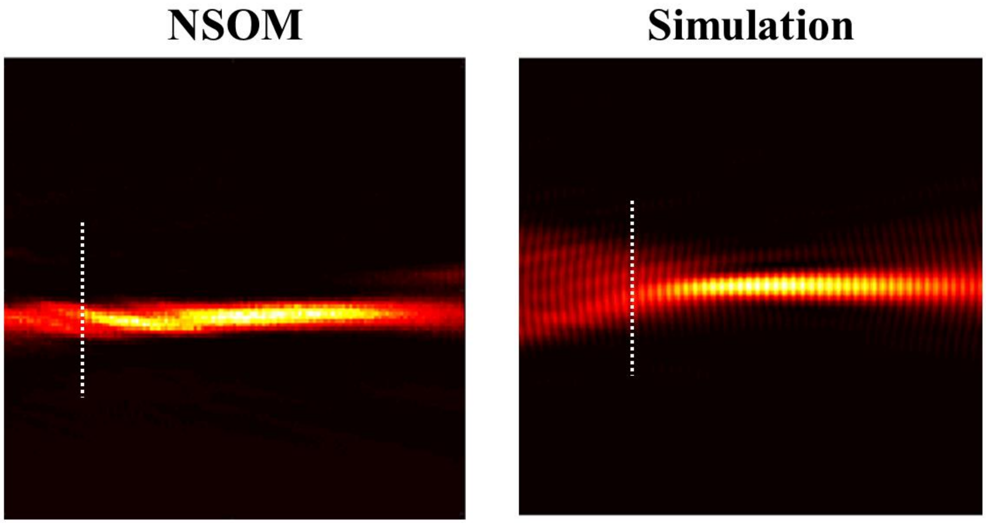

Figure S4. Snapshot describing both similar movies **Movie S1** and **Movie S2** showing the tunable plasmonic lenses under linearly polarized illumination. The left side of screen shows the results obtained from NSOM data, and the right side shows the results of numerical simulation.

Reference

[1] Lee, S.−Y. *et al*. Plasmonic meta-slit: shaping and controlling near-filed focus. *Optica* **2**, 6−13 (2015).
